# Role of Maternal Mindfulness in Longitudinal Mother–Infant Neuroendocrine Reciprocity in an Urban, Low‐Income White Sample

**DOI:** 10.1002/dev.70153

**Published:** 2026-04-14

**Authors:** Kento Suzuki, Heidemarie Laurent

**Affiliations:** ^1^ Department of Human Development and Family Studies The Pennsylvania State University University Park Pennsylvania USA; ^2^ Department of Psychology University of Oregon Eugene Oregon USA

**Keywords:** cortisol, infant, mindfulness, physiological reciprocity, stress co‐regulation

## Abstract

Mother–infant physiological reciprocity plays a crucial role in child well‐being and development. While mindfulness in parenting has been linked to mother–infant stress responses, no research has examined its relationship with developmental trajectories of neuroendocrine reciprocity. This study addresses this gap by examining maternal mindfulness—both alone and in the context of parenting risk and protective factors—in relation to mother–infant cortisol reciprocity over time. Using data from 56 predominantly White, urban, low‐income mother–infant dyads in the United States followed from 3 to 18 months postpartum, we tested maternal mindful parenting at 3 months as a predictor of both (1) within‐session reciprocity of mother and infant cortisol responses to acute stress and (2) across‐session reciprocity of developmental shifts in their cortisol levels from 6 to 18 months. Three‐level hierarchical linear models revealed that higher maternal mindful parenting, greater history of positive parental bonding, and lower parenting stress were associated with more inverse mother–infant cortisol reciprocity across timepoints. In addition, parenting stress moderated the effect of mindful parenting: the inverse relation with reciprocity held for most dyads (60%), though reversed for those with the highest stress levels (top 9%). Findings delineate when and how positive versus inverse reciprocity may be adaptive and underscore the role of mindful parenting in mother–infant psychophysiological stress co‐adaptation.

## 1 Introduction

Reciprocity between mother and infant is recognized as a fundamental cornerstone of children's biological, socioemotional, and cognitive well‐being and adaptation throughout life (Bornstein 2022), and this has been observed across different cultures (Bornstein 2012). The term *reciprocity* typically refers to positive associations between caregiver and offspring behavioral or physiological activity, whereas negative associations are described as *inverse reciprocity* (DePasquale 2020). Mother–infant physiological reciprocity—defined as the ability of two partners to mutually adapt their physiology to each other (Brazelton et al. 1975; Leclère et al. 2014)—has been less examined compared to behavioral reciprocity, and findings regarding the adaptive value of such reciprocity have been mixed (Bernard et al. 2017; van Bakel and Riksen‐Walraven 2008). Although prior research has primarily examined physiological reciprocity over short timeframes within an interactive episode—also called physiological *attunement*—the present study adopts the conceptually broader term without temporal specificity—*reciprocity*—to encompass both moment‐to‐moment mother–infant physiological associations and longer‐term adaptations in dyadic physiological functioning across the first 18 months of infants’ lives (DePasquale 2020; Leclère et al. 2014; Provenzi et al. 2018). Greater mother–infant physiological reciprocity has generally been associated with positive indicators of family functioning, such as greater parental sensitivity and marital satisfaction; at the same time, stronger reciprocity of physiological reactivity between the mother and the infant has also been linked to indicators of harm, especially in high‐risk populations, highlighting that the implications of reciprocity may vary depending on the contextual environment in which dyads are situated (Buhler‐Wassmann and Hibel 2021; DePasquale 2020). In order to determine when and how physiological reciprocity is beneficial, further investigation of mother‐infant reciprocity in relation to known risk and protective factors is needed.

Mindfulness in parenting—the application of present moment, nonjudgmental awareness of unfolding experiences to parenting situations (Duncan et al. 2009)—has been associated with quality of the parent–child relationship and with mothers’ and infants’ physiological stress responses, both on their own and in conjunction with maternal risk characteristics (H. K. Laurent et al. 2017). However, there is as yet no known research examining mindfulness in relation to reciprocity of mother–infant stress physiology. This study aims to address this gap by testing relations between mindfulness in parenting and developmental trajectories of mother–infant cortisol reciprocity across the first year and a half postnatal. In light of evidence that parent–infant stress co‐regulation occurs in context (Buhler‐Wassmann and Hibel 2021), we examine not only direct relations between maternal mindfulness and reciprocity, but also moderation by maternal risk (parenting stress) and protective factors (maternal history of positive parental bonding during childhood) previously shown to impact parent–child physiological co‐regulation (Choi et al. 2021; Doiron et al. 2022).

### 1.1 Caregiver–Infant Reciprocity—Theoretical Frames

Guided by dynamic systems theory, caregiver–infant dyads are conceptualized as interactional systems in which stress regulation patterns emerge from the moment‐to‐moment interplay between partners and cannot be fully explained by the sum of individual attributes (Beeghly and Tronick 2011; Tronick 2007). As such, it is crucial not only to assess the unique contributions of each partner (i.e., parent and infant) but also to capture how one partner's responses dynamically influence the other's in real time—often referred to as *attunement* (Beebe et al.^2010^; Beeghly and Tronick 2011). As noted above, attunement is typically distinguished from *reciprocity*, with the former conceptualized as a subdivision of the latter that specifically focuses on short‐term, moment‐to‐moment coordination, often in response to a specific task or context (DePasquale 2020; Leclère et al. 2014; Provenzi et al. 2018). This framework has been applied to the examination of mother–infant stress co‐regulation specifically at the physiological level, with research exploring not only individual patterns of physiological reactivity to stressors within the dyad, but also the degree to which these physiological responses are linked and mutually influenced (Atkinson et al. 2016).

Further understanding of how and why co‐regulation occurs comes from attachment theory, in which the caregiver–infant relationship is understood as a primary developmental context for the regulation of stress physiology (Bowlby 1969; Hostinar et al. 2014). Infants communicate their internal states and needs to their co‐regulatory partners through emotional bids—such as crying, gazing, and proximity‐seeking—to which caregivers respond with varying sensitivity (Bowlby 1969). These caregiver responses play a critical role in shaping infants’ reactivity to and recovery from stress at both behavioral and physiological levels (Feldman 2007). In turn, infants’ responses influence caregivers’ subsequent behavioral and physiological engagement in the dyadic regulation process (Guo et al. 2015). Through such repeated interactions—especially during moments of distress—infants begin to internalize expectations about how their caregivers will respond. In this way, repeated infant‐caregiver interactions result in embedding of dynamic physiological co‐regulatory patterns by which stress response systems develop (Gunnar and Donzella 2002). Building on this foundational notion, several theoretical frameworks emphasize the significance of physiological reciprocity in caregiver–child relations to understand biological embedding of learned adaptation to stress (Atkinson et al. 2024), and activity of the hypothalamic‐pituitary‐adrenal (HPA) axis is one of the most intensively studied targets in this domain (H. K. Laurent et al. 2017).

### 1.2 Physiological Reciprocity in Caregiving Dyads

The HPA axis is a critical part of the human stress response system, functioning as a neuroendocrine cascade that results in the release of cortisol, a glucocorticoid hormone that mobilizes stress‐related neural and somatic processes to manage threat (Smith and Vale 2006). This system demonstrates both normative diurnal fluctuations—characterized by high cortisol levels in the morning and lower levels at night—and acute reactivity in response to stress followed by recovery (Markovic et al. 2011). Literature suggests that reciprocity of the activation of the HPA axis between the mother and the infant—typically measured through salivary cortisol—is associated with aspects of positive parenting, such as maternal sensitivity and physical contact (Atkinson et al. 2013; Cristóbal Cañadas et al. 2022). On the other side of the reciprocity spectrum, studies have linked divergent mother‐infant cortisol patterns to negative relationship indicators, such as disrupted maternal communication and attachment disorganization (Crockett et al. 2013; Nofech‐Mozes et al. 2020). However, findings in the opposite direction suggest that cortisol reciprocity does not always characterize positive family environments and may foster risk transmission. For example, there is evidence that mothers with risk characteristics, such as war exposure or maltreatment, show stronger cortisol associations with their infants that, in turn, predict child behavioral problems (Halevi et al. 2017; Hibel et al. 2019), and a recent mixed human/rodent study demonstrated paths from early life adversity to child behavior difficulties via mother–infant cortisol linkage (Perry et al.^2020^). In a similar vein, our previous research in a lower‐income sample revealed that maternal factors indicating greater risk—such as lower family resources and social support satisfaction, or higher parenting stress—predicted higher mother‐infant cortisol reciprocity across the first few years of postnatal development (H. K. Laurent et al. 2021).

### 1.3 Contextual Considerations in Physiological Reciprocity

Such inconsistencies in findings on mother‐infant cortisol reciprocity may partly reflect the fact that similar physiological reactivity patterns can have different implications across contexts (Boyce and Ellis 2005). According to the biological sensitivity to context theory, heightened HPA reactivity to stress can function as either a risk‐enhancing or protective factor depending on the child's environmental conditions, including household income. Specifically, greater stress reactivity may support adaptive functioning in low‐risk, high‐resource environments but predict developmental difficulties in higher‐risk contexts characterized by threat or unpredictability (Armstrong‐Carter and Telzer 2022; Obradović et al. 2016). As such, the meaning of parent–infant physiological activation and reciprocity in higher‐risk samples may diverge from that observed in lower‐risk populations (Atkinson et al. 2016), warranting further investigation in lower‐SES populations representing moderate environmental risk to determine whether heightened reciprocity—like reactivity itself—is a liability in this context.

Another relevant distinction concerns when reciprocity is measured and how it is operationalized (Hibel et al. 2015; H. K. Laurent et al. 2021). Infant physiological responses to psychosocial stress and their relationships with maternal parenting characteristics show variable trends during the first few years of life (H. K. Laurent et al. 2016). Such variability reflects rapid neurophysiological development during infancy and infants’ heightened sensitivity to caregiving environments (Gilmore et al. 2012; Troller‐Renfree et al. 2020). Although patterns of physiological regulation vary depending on caregiving quality and may represent adaptive responses to the infant's environment (Del Giudice et al. 2011; Gunnar and Donzella 2002), infants generally develop strategies to self‐regulate their stress responses over the first 18 months of life through repeated parent–infant co‐regulatory interactions (Beeghly and Tronick 2011; DiCorcia and Tronick 2011). This highlights the importance of examining parent–infant physiological reciprocity not only cross‐sectionally within short timeframes but also longitudinally, to understand how mothers adapt to and support developmental shifts in their infants’ stress physiology (Bell 2020; Davis et al. 2018). This approach is particularly important given evidence that sensitive parenting in early infancy is associated with developmental downregulation of infant HPA axis activity over the first 15 months after birth (Gunnar et al. 1996; Gunnar and Cheatham 2003). A nuanced approach that distinguishes reciprocity across multiple time scales could provide a more comprehensive understanding of this phenomenon.

Longitudinal studies of mother‐infant cortisol reciprocity remain scarce, particularly those examining reciprocity both within and across episodes during infancy. For instance, one of the few existing studies examined how within‐episode mother–infant cortisol attunement varied across the first 2 years of life (Hibel et al. 2015). However, it did not address whether mothers’ physiological functioning shifted in ways that aligned with their infants’ developmental shifts in HPA axis activation to stress, leaving long‐term patterns of developmental reciprocity largely unexplained. Toward addressing this gap, we have proposed separating reciprocity of mother‐infant cortisol responses *within a given stress episode*—attunement—from similarity (or dissimilarity) in *developmental shifts* of stress system activation across infancy. Previous research with the current sample provided evidence for distinct effects of dyad risk characteristics at different levels of reciprocity (H. K. Laurent et al. 2021). In line with this framework, the present study addresses the extent to which mother and infant trajectories of cortisol change in parallel across a 1‐year period of early development, over and above concurrent associations between their cortisol levels in response to stress at specific timepoints.

In addition to distinguishing levels of reciprocity, identifying behavioral correlates may clarify when and how reciprocity is adaptive. Among maternal characteristics shaping mother‐infant relationships, maternal mindfulness has received increasing attention (Ahemaitijiang et al. 2021). Mindfulness—defined as “paying attention in a particular way: on purpose, in the present moment, and nonjudgmentally” (Kabat‐Zinn 1994)—is postulated to promote parenting qualities, including parental sensitivity, and empirical research supports this assertion (Campbell et al. 2017). Meta‐analytic findings indicate that cultivating mindfulness reduces stress/distress in parents (Anand et al. 2023) while promoting adaptive HPA axis activation patterns, including lower stress‐response cortisol levels and more negative diurnal cortisol slopes (Vargas‐Uricoechea et al. 2024). Although often conceptualized as an intrapersonal capacity, mindfulness is increasingly studied in relational contexts, including parenting across various demographic groups (Kabat‐Zinn 1990; Mera et al. 2023; Yan et al. 2021). Specifically, mindfulness may influence interpersonal interactions by fostering sustained attention toward others, enhancing prosocial attitudes and behaviors, and reducing stress and reactivity in interactions (Khoury et al. 2023).

### 1.4 Mindfulness in Parenting and Caregiver–Infant Reciprocity

In parenting contexts, mindful parenting—the application of present moment, nonjudgmental awareness of unfolding experiences to parenting situations—has garnered significant attention (Anand et al. 2023). As laid out by Duncan et al. (2009), mindful parenting consists of five key components: (1) listening with full attention, (2) nonjudgmental acceptance of self and child, (3) self‐regulation in the parenting relationship, (4) emotional awareness of self and child, and (5) compassion for self and child. As outlined in the Mindful Parenting Processes Model, mindful parenting strengthens the quality of parent‐child relationships through intra‐ and interpersonal processes, including enhanced emotional reciprocity whereby the parent brings intentional awareness to their child's mental state in ways that allow them to “feel felt” (Townshend 2016). Consistent with this model, mindful parenting has been empirically linked to positive parenting practices and more secure maternal‐infant attachment (Fernandes et al. 2021, 2022). Its positive impacts are further supported by associations with other constructs emphasized in attachment theory, such as reflective functioning (Falkenström et al. 2014; Huynh et al. 2024; Shaver et al. 2007), and have also been demonstrated among high‐risk dyads, including those from low‐income families (Parent et al. 2014; Rivera et al. 2022).

Despite such evidence that mindfulness can enhance mother‐infant reciprocity at the behavioral level, little is known about whether these effects extend to and/or are scaffolded by physiological reciprocity. In prior work, we found associations between maternal mindful parenting and both maternal and infant cortisol responses during a dyadic stressor task. Specifically, higher mindful parenting predicted steeper maternal cortisol recovery slopes—indicating faster downregulation of HPA axis activity following the stressor—and life stress moderated the effect of mindful parenting on later mother and infant cortisol (H. K. Laurent et al. 2017). However, that study did not assess the coordination of partners’ responses. To our knowledge, only one study has addressed maternal mindfulness in relation to mother–infant physiological reciprocity. In a healthy low‐risk sample, higher maternal dispositional mindfulness—reflecting one's general ability to be mindful in daily life, as opposed to momentary state mindfulness or mindfulness cultivated through formal practice (Burzler and Tran 2022)—was associated with greater autonomic coordination (RSA cross‐correlations) during free play at 3 months postpartum (Passaquindici et al. 2024). Thus, although there is emerging evidence linking maternal mindfulness with mother and infant stress physiology, no research has tested implications of mindful parenting for the coordination of mother and infant neuroendocrine stress regulation.

To gain further insight into whether and how mindfulness can benefit mother–infant stress regulation, it is crucial both to test this association across multiple levels of reciprocity and in relation to other known protective and risk factors. Factors known to impact parent‐child relationship quality and stress physiology exist along a continuum from risk to protection, with the mother's levels of parenting stress on the former side and her own history of receiving parental care on the latter (Atkinson et al. 2024). For example, parenting stress has been associated with maladaptive parenting behaviors (McMahon and Meins 2012) and reduced brain‐to‐brain synchrony between parent and infant during joint activities (Azhari et al. 2019). In contrast, the history of parental care—the experience of parental attitudes such as affection, emotional warmth, empathy, and closeness expressed by one's primary caregivers during upbringing—has been linked with more adaptive parent‐child physiological co‐regulation and parenting behaviors (Parker et al. 1979). For instance, a greater history of parental care has been associated with reduced distress during the transition to parenthood and increased positive responsiveness toward one's infant (Grant et al. 2012; Madden et al. 2015).

Such risk/protective factors may also moderate links between mindful parenting and mother–infant reciprocity, as mindfulness effects are often dependent on aspects of the parenting context. For example, our previous study showed that associations between maternal mindfulness in parenting and their own cortisol depended on levels of life stress (H. K. Laurent et al. 2017). Similarly, maternal depressive symptoms have been associated with lower bonding quality among parents with lower levels of mindfulness but not among those with higher levels of mindfulness (Hicks et al. 2018). These findings suggest that the pathways from maternal mindfulness to cortisol and/or co‐regulation with their infant may be influenced by the risk context in which the dyad is operating, offering another layer of insight into what constitutes an “adaptive” physiological stress response profile for a given dyad.

### 1.5 The Current Study

As outlined above, there is as yet no known research examining mindfulness in relation to developmental trajectories of mother–infant physiological reciprocity, either in isolation or in the context of other maternal risk and protective factors (H. K. Laurent et al. 2021). This study aims to address this gap by examining maternal mindfulness, history of positive parental bonding, and parenting stress in relation to mother–infant cortisol reciprocity over time in a higher‐risk, low‐income sample. We examined maternal mindfulness as a focal predictor of mother–infant cortisol reciprocity and assessed whether this relationship was moderated by parenting stress and history of positive parental bonding. Reciprocity was considered at the levels of both (1) within‐session cortisol trajectory associations and (2) coordination of relative shifts in cortisol levels across developmental periods. While recognizing that infants develop within relational systems involving multiple significant caregivers and socialization agents, we focused on mothers, given that mothers continue to constitute the primary caregivers during infancy (Mchale 2007).

Based on the literature reviewed above—including our own previous findings—and conceptualizing the current lower‐SES sample as representing a generalized risk context, we hypothesized that (1) higher levels of maternal mindful parenting and positive parental bonding at 3 months postpartum would be inversely associated with mother–infant cortisol reciprocity at both within‐ and across‐episode levels (i.e., more negative association of mother and infant cortisol changes both within‐episode and across‐episode); (2) maternal parenting stress would be positively associated with mother–infant cortisol reciprocity at both within‐ and across‐episode levels; and (3) effects of maternal mindful parenting on cortisol reciprocity would be moderated by parenting stress at both within‐ and across‐episode levels. Given the absence of prior research informing contextual effects of mindfulness in parenting on reciprocity, we did not hypothesize a direction of effect.

## 2 Method

### 2.1 Transparency and Openness

We report how we determined our sample size, all data exclusions (if any), all manipulations, and all measures in the study, and we follow JARS (Appelbaum et al. 2018). All data, analysis code, and research materials are available from the second author upon request. Data were analyzed using the HLM 7.0 program (Raudenbush et al. 2016). This study's design and its analysis were not preregistered.

### 2.2 Participants

Participants were drawn from a longitudinal study investigating mother–infant stress regulation, which included assessments at 3, 6, 12, and 18 months postpartum. Recruitment focused on mothers from the Women, Infants, and Children (WIC) program and other community agencies supporting low‐income families in a mid‐sized city in the northwest region of the United States. Fathers were not included in the present study because pilot work conducted with the same community organization indicated that fathers were often inconsistently involved in the child's life, if not entirely absent. We therefore focused on mothers, who appeared to represented a more stable and consistent influence in infants’ daily caregiving environments. Eligibility criteria included English fluency, having an infant younger than 12 weeks, and planning to remain in the area until the child reached 18 months of age. In the current study sample, 76.9% of participants identified as White, with 9.9%, 3.3%, 2.2%, and 3.3% identifying as Latina, African American, Native American, and Asian American, respectively. The average age of the sample was 28.32 years, and only 8.8% of participants had a 4‐year college degree or higher. In terms of household income, 31.9% reported earning less than $5000, and 82.5% reported earning less than $50,000 (detailed demographic information can be found in H. Laurent 2017). Of the 91 mothers initially enrolled in the study, 54 completed the questionnaire portion of all four assessments (*n* = 76, 61, and 53 dyads with complete cortisol data at 6, 12, and 18 months, respectively). Mothers who discontinued participation were generally characterized by higher levels of initial risk factors, such as being younger, having lower income, and exhibiting higher depressive symptoms (H. Laurent 2017). The sample size was not dictated by a priori power analysis but was constrained by the funding and temporal constraints of the award supporting this research. To be included in analysis, a dyad had to have complete data for the T1 variables listed below, as well as mother and infant cortisol values from at least one session; this resulted in a sample of 56 dyads for the current study. A comparison of those included versus those not included revealed that the only study variable on which groups differed was history of parental care, with mothers included in the current sample reporting a higher history of parental care than those who were not included, *t*(85) = 2.26, *p* = 0.026, *M* = 23.83 versus 27.86.

### 2.3 Procedures

All procedures were reviewed and approved by the University of Oregon Institutional Review Board and adhere to the US Federal Policy for the Protection of Human Subjects. Prior to participating, all participants provided written informed consent. At 3 months postnatal, mothers completed self‐report questionnaires, and during the latter three assessments, they engaged in dyadic stress‐inducing laboratory tasks with their infants. These tasks included the Still Face Procedure at 6 months (Toda and Fogel 1993), the Strange Situation at 12 months (Ainsworth and Wittig 1969), and the Maternal Separation and Stranger Approach episodes from the Laboratory Temperament Assessment Battery at 18 months (Goldsmith and Rothbart 1991). The ecological validity of these stress tasks has been well‐established. For instance, parental withdrawal in the Still Face Procedure has been considered as reflecting aspects of everyday parenting interactions, especially among families in western contexts (Broesch et al. 2022; Mesman et al. 2009). The Strange Situation, developed from naturalistic observations in Uganda and Baltimore, has been shown to elicit infant behaviors consistent with those observed at home (Ainsworth et al. 1978). Similarly, parents describe their children's responses in the Maternal Separation and Stranger Approach episodes as representative of typical behavior outside the lab (Lo et al. 2015). Importantly, all three of the paradigms used in the current study have been empirically validated as effective stressors in mother–infant dyadic settings (Gunnar et al. 2009). During each stress session, mothers and infants provided four saliva samples for cortisol assays, as described below. Sessions were scheduled in the afternoon to control for diurnal cortisol variations, and a presession survey collected information on potential confounding factors, such as recent food or drink consumption and illness. If key conditions were violated, the sessions were rescheduled. Reciprocity modeling was based on data from the latter three stress‐task sessions involving cortisol sampling, while parenting factors—maternal mindfulness, parenting stress, and history of parental care—were derived from the first assessment to offer prospective prediction.

### 2.4 Measures

#### 2.4.1 Interpersonal Mindfulness in Parenting–Infant Version (IMP–I)

This measure represents an adaptation of the Interpersonal Mindfulness in Parenting measure (Duncan 2007; Duncan et al. 2009) for the infant stage of development and comprises 27 items rated on a 5‐point scale from *never true* to *always true*. Representative items include “I pay close attention to my baby when we are spending time together,” “I notice how changes in my baby's mood affect my mood,” “When things I try to do as a parent do not work out, I can accept them and move on,” and “I often react negatively when my baby fusses or cries” (reverse scored). A mean score was computed for analysis. Internal consistency for the scale was acceptable (Cronbach's alpha: 0.81). Total mean IMP–I scores ranged from 3.17 to 4.77 (*M *= 4.16, *SD *= 0.36). The IMP–I was originally developed based on confirmatory factor analyses conducted on a predominantly White, middle‐aged parents of adolescents and later validated for parents of infants (Duncan 2007).

#### 2.4.2 Five Facet Mindfulness Questionnaire

The Five Facet Mindfulness Questionnaire (FFMQ) measures general dispositional mindfulness with 39 items rated on a 5‐point scale from *never or very rarely true* to *very often or always true*. Example items include “I find it difficult to stay focused on what's happening in the present” (reverse‐scored) and “When I have distressing thoughts or images, I just notice them and let them go.” Cronbach's alpha for the total scale was 0.90. A mean score was computed for analysis. Total mean FFMQ scores ranged from 2.70 to 4.69 (*M *= 3.63, *SD *= 0.52). The FFMQ was originally developed based on confirmatory factor analyses conducted on a predominantly White, young adult female population (Baer et al. 2006).

#### 2.4.3 Parental Bonding Instrument

Parental Bonding Instrument (PBI) evaluates perceived parenting styles—care and overprotection—of their mother and father up to the age of 16, with 25 items rated on a 4‐point scale from *very unlike* to *very like*. Only the 11 care items were used for the analysis of this study as a parental protective factor. Example care items include “Spoke to me with a warm and friendly voice” and “Did not help me as much as I needed” (reverse‐scored). A sum score was computed for each caregiver and averaged across caregivers to index total care experienced. Cronbach's alpha for the total subscale was 0.95. Total mean PBI Care subscale scores ranged from 3.00 to 36.0 (*M *= 26.2, *SD *= 8.34). Based on the scale developers’ cutoff for mothers (summed score < 27), approximately half the current sample (48%) reported experiencing low levels of care (Parker et al. 1979).

#### 2.4.4 Parental Stress Scale

The Parental Stress Scale (PSS) includes 18 items to assess thoughts and feelings related to parenting that contribute to stress, rated on a scale from *strongly disagree* to *strongly agree* for such statements as “Caring for my child sometimes takes more time and energy than I have to give” and “If I had to do over again, I might decide not to have children.” The measure has been found to relate to general measures of stress and to measures of relevant emotions and role satisfaction. Cronbach's alpha for the total scale was 0.80. A sum score was computed for analysis. Total summed PSS scores ranged from 19.0 to 55.0 (*M *= 31.8, *SD *= 7.24) (Berry and Jones 1995).

### 2.5 Cortisol

As previously described (H. Laurent 2017), saliva samples were collected from mothers and infants at four time points across the session to measure stress responsivity and recovery: (1) shortly after arriving at the laboratory, (2) immediately after the peak stressor, (3) 20 min after the onset of the peak stressor, and (4) 30 min following the third sample. Mothers provided samples via passive drool using Salimetrics Saliva Collection Aids, while infant samples were collected using Salimetrics Infant/Child Swabs. All saliva samples were assayed in duplicate using the Salivary Cortisol Enzyme Immunoassay (Salimetrics, Carlsbad, CA), following the manufacturer's protocol without modifications. Each test required 25 µL of saliva, with a sensitivity lower limit of 0.007 µg/dL and a standard curve range of 0.012–3.0 µg/dL. The intra‐assay coefficient of variation was on average < 10%, and the inter‐assay coefficient of variation was on average < 15%. To address data skewness, cortisol scores were natural log‐transformed prior to analysis. Several control variables—such as session start time, medication or substance use, sleep/wake patterns, illness, body mass index, and infant feeding during the session—were evaluated but did not consistently impact cortisol levels for either mothers or infants. As a result, these variables were excluded from further analyses.

### 2.6 Analytic Strategy

To model multiple levels of mother–infant cortisol reciprocity, a 3‐level model was fit using the HLM 7 program. This approach separates variability into within‐dyad, within‐episode (Level 1), within‐dyad, across‐episode (Level 2), and across‐dyad (Level 3) levels. To represent mothers’ responsiveness to their infants’ stress activation, maternal cortisol served as the outcome, and infant cortisol was added to predict changes in mother cortisol levels. Model terms were allowed to vary across episodes and dyads to test random effects.

At Level 1, infant cortisol (each sample value) was entered as a grand mean‐centered predictor of concurrent mother cortisol; this term reflected cortisol attunement, capturing within‐episode reciprocity in the sense of similar reactivity/recovery profiles to a given stressor. At Level 2, infant cortisol (mean sample value from each stress episode) was entered as a group mean‐centered predictor of mother cortisol; this term reflected across‐episode developmental reciprocity in the sense of similar relative shifts in HPA activation to stressors from 6 to 18 months. Maternal characteristics of interest—that is, mindfulness in parenting, history of positive parental bonding, and parenting stress—were entered as Level 3 predictors of between‐dyad variability in reciprocity terms to address primary research questions. The example model below illustrates the test for a main effect of mindfulness in parenting on within‐episode and across‐episode mother–infant cortisol reciprocity:

Level 1: Mother Cortisol = π*_0_ +*π*_1_* (Infant cortisol at same sample) + error
Level 2: π*_0_* = β *_00_* + β *_01_* (Infant mean cortisol at same episode) + error
π*_1_* = β *_10_* + error
Level 3: β *_00_* = γ*_000_* + γ*_001_* (T1 IMP score) + error
β *_01_* = γ*_010_* + γ*_011_* (T1 IMP score) + error
β *_10_* = γ*_100_* + γ*_101_* (T1 IMP score) + error

In addition, to examine whether developmental physiological downregulation patterns were observed among infants in the current sample, we conducted a repeated‐measures ANOVA and Bonferroni‐corrected paired *t*‐tests to test whether group‐mean‐centered infant cortisol values changed meaningfully over time.

## 3 Results

Table 1 presents the correlations among the variables examined in the study.

**TABLE 1:** Means, standard deviations, and correlations with confidence intervals.

Variable	M	SD	1	2	3	4	5	6	7	8	9
(1) Infant cortisol at 6 months	−1.46	0.60									
(2) Infant cortisol at 12 months	−1.56	0.85	0.17 [−0.08, 0.40]								
(3) Infant cortisol at 18 months	−1.95	0.52	0.19 [−0.08, 0.44]	0.47** [0.23, 0.66]							
(4) Maternal cortisol at 6 months	−2.28	0.42	0.15 [−0.07, 0.36]	−0.09 [−0.33, 0.16]	−0.21 [−0.46, 0.06]						
(5) Maternal cortisol at 12 months	−2.26	0.68	0.04 [−0.21, 0.28]	0.21 [−0.04, 0.43]	−0.18 [−0.42, 0.10]	0.15 [−0.10, 0.38]					
(6) Maternal cortisol at 18 months	−2.49	0.63	0.09 [−0.19, 0.35]	−0.08 [−0.34, 0.20]	−0.09 [−0.35, 0.18]	0.25 [−0.02, 0.49]	0.19 [−0.09, 0.43]				
(7) FFMQ at 3 months	3.63	0.52	0.01 [−0.21, 0.23]	−0.02 [−0.27, 0.23]	0.02 [−0.25, 0.29]	−0.05 [−0.27, 0.17]	−0.03 [−0.28, 0.23]	0.05 [−0.23, 0.31]			
(8) IMP−I at 3 months	4.16	0.36	0.18 [−0.05, 0.39]	0.18 [−0.08, 0.42]	0.11 [−0.18, 0.37]	0.06 [−0.17, 0.28]	−0.08 [−0.33, 0.18]	−0.20 [−0.45, 0.08]	0.26* [0.05, 0.46]		
(9) PBI Care at 3 months	26.24	8.34	−0.05 [−0.27, 0.18]	0.09 [−0.17, 0.34]	−0.06 [−0.33, 0.22]	0.05 [−0.17, 0.27]	0.17 [−0.09, 0.41]	0.05 [−0.22, 0.32]	0.04 [−0.18, 0.25]	−0.01 [−0.23, 0.21]	
(10) PSS at 3 months	31.77	7.24	−0.09 [−0.31, 0.14]	−0.11 [−0.35, 0.15]	−0.02 [−0.29, 0.25]	−0.04 [−0.26, 0.19]	−0.13 [−0.37, 0.13]	−0.07 [−0.34, 0.20]	−0.30** [−0.48, −0.09]	−0.56** [−0.69, −0.39]	−0.15 [−0.35, 0.06]

*Note:* M and SD are used to represent mean and standard deviation, respectively. Values in square brackets indicate the 95% confidence interval for each correlation.

Abbreviations: FFMQ = Five Facet Mindfulness Questionnaire; IMP–I = Inventory of Mindfulness in Parenting–Infant Version; PBI Care = Parental Bonding Instrument Care subscale; PSS = Parenting Stress Scale.

*indicates *p* < 0.05; **indicates *p* < 0.01.

### 3.1 Baseline Model

First, a baseline 3‐level model was fit to determine whether there was evidence of normative mother–infant cortisol reciprocity and/or significant variability in reciprocity parameters that could be explained by maternal characteristics. This model did not reveal normative sample‐wide reciprocity (*γ*_100_ = 0.026, *SE = *0.044 within‐episode and *γ*_010_ = −0.019, *SE = *0.133 across‐episode, both *ns*) but it did show evidence of variability in these parameters across dyads (*χ* ^2^[46] = 63.76, *p *= 0.042 for within‐episode and *χ* ^2^[46] = 71.12, *p *= 0.010 for across‐episode), supporting the addition of explanatory predictors.

### 3.2 Explanatory Models

The first explanatory model tested the main effect of mothers’ T1 IMP–I scores, controlling for their T1 FFMQ scores, to determine whether there was a unique effect of mindfulness in parenting—over and above general dispositional mindfulness—on mother–infant cortisol reciprocity patterns. We found a significant negative effect of T1 IMP–I on the Level 2 across‐episode reciprocity index, indicating that mothers higher in mindful parenting showed more inverse developmental reciprocity to their infants—that is, when the infant increased in cortisol from one session to the next, this tended to be accompanied by a decrease in the mother's cortisol (Table 2, top Panel 1). This model explained 16.3% of the between‐dyad variance in across‐episode cortisol reciprocity.

**TABLE 2:** Model results for multilevel mother–infant cortisol attunement.

Predictor	Intercept (mean mother cortisol)		Across‐episode attunement (effect of infant cortisol at concurrent session)		Within‐episode attunement (effect of infant cortisol at concurrent sample)	
	*γ*	*p*	*γ*	*p*	*γ*	*p*
(1) T1 IMP–I	−0.056	0.494	**−0.265**	**0.035**	−0.044	0.421
T1 FFMQ	0.086	0.423	−0.101	0.388	0.067	0.104
(2) T1 IMP–I	−0.032	0.683	**−0.300**	**0.028**	−0.023	0.664
T1 PBI Care	0.240	0.014	**−0.426**	**0.037**	0.002	0.974
(3) T1 IMP–I	−0.076	0.448	**−0.185**	**0.032**	−0.015	0.799
T1 PS	−0.083	0.480	**0.248**	**0.022**	0.034	0.402
T1 IMP–I × PS	−0.055	0.627	**0.348**	**0.001**	−0.023	0.559

*Note*. γ’s represent standardized coefficients. Significant effects (*p* < 0.05) on attunement parameters are highlighted in bold.

Abbreviations: IMP–I = Inventory of Mindfulness in Parenting—Infant Version; FFMQ = Five Facet Mindfulness Questionnaire; PBI Care = Parental Bonding Instrument Care subscale; PS = Parenting Stress.

Next, we tested the effect of mothers’ T1 IMP–I scores in the context of baseline promotive (PBI Care) and risk (PS) factors in the parenting relationship, considering both main effects and interactions. The first model examining positive parental bonding history showed a significant main effect of T1 PBI Care alongside IMP–I in the same (negative) direction on across‐episode reciprocity. This indicated that mothers’ history of receiving care from their parents, and mindful parenting of their own infants, each contributed to predicting more inverse developmental cortisol reciprocity (Table 2, middle Panel 2). The interaction of mindful parenting with positive parental bonding was not significant. The final main effects model explained 37.4% of the between‐dyad variance in across‐episode cortisol reciprocity.

**FIGURE 1:**
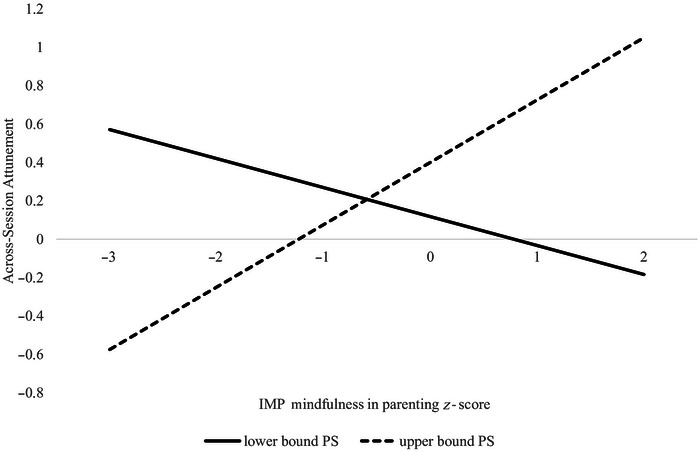
Moderated effect of maternal mindfulness in parenting on mother–infant developmental cortisol attunement by parenting stress.

By contrast, the model examining parenting stress showed a positive effect of T1 PS on across‐episode reciprocity, though this effect was conditioned by a significant positive interaction with T1 IMP–I (Table 2, lower Panel 3). A region of significance calculator (Preacher et al. 2006) was used to interpret the interaction; this revealed that for most of the sample (bottom 60% of PS scores), mindful parenting predicted more inverse developmental reciprocity as suggested above, but for the highest‐stress portion of the sample (top 9% of PS scores) mindful parenting predicted more positive coordination of mother and infant cortisol shifts across episodes (Figure 1). The final interactive effect model explained 26.5% of the between‐dyad variance in across‐episode cortisol reciprocity. There were no significant effects detected for between‐dyad differences in within‐episode (Level 1) mother‐infant reciprocity.

Lastly, a repeated‐measures ANOVA revealed a significant effect of session on group‐mean‐centered infant cortisol, *F*(1.71, 80.5) = 12.0, *p* < 0.001, *η*(G^2^) = 0.20. Bonferroni‐corrected paired *t*‐tests indicated no difference between 6 and 12 months, *t*(55) = 1.01, *p* = 0.95, but significantly lower cortisol at 18 months compared with both 6 and 12 months, *t*(47) = 5.26, *p* < 0.001, and 12 months, *t*(47) = 4.22, *p* < 0.001, respectively. (Figure 1)

## 4 Discussion

In this study, we aimed to elucidate the role of mindful parenting in reciprocity of mother–infant cortisol across early development. Toward this aim, we considered both direct relations between mindful parenting and reciprocity and moderation by maternal risk (parenting stress) and protective factors (history of positive parental bonding) across the first year and a half postnatal. Overall, the results supported study hypotheses: mindfulness in parenting was associated with more inverse mother–infant cortisol reciprocity across early development, which paralleled effects of factors known to promote positive parenting—that is, history of high parental care and low parenting stress. More specifically, among dyads in which mothers reported higher levels of mindful parenting, mothers showed relatively higher physiological activation on occasions when their infants exhibited lower HPA axis activation than at other occasions. There was also evidence for a contextual effect of mindfulness in parenting, with stronger inverse reciprocity effects at lower levels of parenting stress but the opposite (i.e., positive reciprocity) at the highest levels of parenting stress. This adds to knowledge of how mindful parenting may shape parent–infant relationships at the level of stress physiology, specifically suggesting that HPA activation shifts in the infant may be accompanied by opposing shifts in the mother under more favorable parenting conditions. In light of recent empirical evidence (Vernon and Moretti 2024) linking mindful parenting to more adaptive parental emotion regulation—measured via parent self‐report of emotion regulation in the context of the parent–child relationship—these findings may imply that mindfulness enables mothers to support their infants’ stress regulation by remaining regulated themselves. Although the present study did not directly assess maternal emotion regulation and instead focused on dyadic cortisol reciprocity, this prior work may help shed light on processes that underlie the inverse pattern of mother–infant cortisol reciprocity observed in the present study. All of these effects were found for across‐episode but not within‐episode of cortisol reciprocity, underlining the importance of a multilevel approach to describe the phenomenon.

### 4.1 Mindfulness in Parenting and Caregiver–Infant Reciprocity

Even though there has been no empirical study of mindfulness and developmental trajectories of cortisol levels, evidence suggests that higher levels of mindfulness are generally associated with more adaptive patterns of HPA axis activation, as mentioned earlier (Vargas‐Uricoechea et al. 2024). This evidence, therefore, offers a broader context for interpreting the inverse across‐episode cortisol reciprocity associated with higher maternal mindfulness observed in the current study. In addition, our previous study showed that higher mindful parenting predicted steeper maternal cortisol recovery slopes and lower infant cortisol levels in response to an acute stressor under normative (lower) life stress conditions, suggesting that mindfulness may facilitate more adaptive parent–infant dyadic adrenocortical responses when facing challenges (H. K. Laurent et al. 2017). Although we are not able to deduce causal relationships based on the study design, our finding of an inverse relationship between mindfulness in parenting and across‐episode mother–infant cortisol reciprocity may help us understand how more adaptive stress responding is transmitted from mindful parents to their children—that is, through particular patterns of reciprocal dyadic physiological activation across multiple stress response/recovery episodes. The interpretation that the inverse reciprocity pattern associated here with maternal mindfulness can be considered adaptive is further bolstered by its alignment with another known protective factor—that is, the mother's own history of positive parental bonding, specifically in the form of parental care. More broadly, these results are consistent with theoretical models proposing that dyadic physiological reciprocity may represent one pathway through which mindfulness in parenting could promote more adaptive infant stress regulation (Sansone 2024; Skoranski et al. 2019). Future research involving parental mindfulness interventions could help to test these ideas more fully.

### 4.2 Differential Implications of Caregiver–Infant Reciprocity Across Contexts

As introduced earlier, biological sensitivity to context theory outlines how profiles of stress‐related physiological activation—and their implications—vary by context (Boyce and Ellis 2005). Specifically, heightened HPA activation to stress can confer adaptive advantages in low‐risk, supportive contexts but may become deleterious in high‐risk or adverse environments. The present study extends this perspective by suggesting that sensitivity may also be expressed at the dyadic level, in the degree to which mothers’ and infants’ physiological responses are reciprocated over time, offering new insight into how sensitivity profiles operate within caregiving relationships. In addition, the current findings add to growing knowledge of the conditions under which positive versus inverse reciprocity may be considered adaptive in parenting dyads. As noted previously, research in low‐risk samples has generally identified a positive association between mother–infant cortisol reciprocity and positive parenting factors such as maternal sensitivity and positive affect (Atkinson et al. 2013; van Bakel and Riksen‐Walraven 2008), whereas in families with contextual risk factors, positive dyadic behavioral patterns have been associated with lower mother–infant cortisol reciprocity (Pratt et al. 2017), as was the case in the current study. This pattern echoes and adds to our previous finding that protective maternal factors—family resources and social support satisfaction—predicted lower mother–infant cortisol reciprocity within stress episodes (H. K. Laurent et al. 2021). Taken together, this work supports the idea that for parent‐infant dyads in challenging contexts such as lower SES, the parental role may involve anticipating and responding to environmental challenges to help keep the infant's stress‐response systems calm, and that this may manifest as a “purposive” lack of dyadic physiological reciprocity (Atkinson et al. 2016). Although the current operationalization of reciprocity does not specify directional effects, this pattern likely reflects reciprocal mother–infant influences giving rise to diverging HPA activation profiles over the course of these early years. For example, mindful parents’ relative upregulation of their own HPA axis engagement during stressful interactions may function to support their child's normative course of HPA axis downregulation—a hypothesis consistent with prior evidence that higher maternal HPA axis reactivity is associated with greater parental sensitivity, including an enhanced ability to differentiate among different types of infant cries (Stallings et al. 2001). Moreover, greater dispositional mindfulness has been linked to higher acute cortisol responses to stress (Gallistl et al. 2024). In combination, these findings from previous studies, together with the observed association between mindful parenting and inverse across‐episode reciprocity as well as the finding that infants in the current sample on average demonstrated developmental downregulation of HPA axis activity—especially from 12 to 18 months postpartum—provide converging support for this hypothesis.

### 4.3 Maternal Parenting Stress and Caregiver–Infant Reciprocity

On the other side of the risk‐protective factor continuum, we observed that parenting stress was associated with positive mother–infant cortisol reciprocity across episodes, aligning broadly with evidence that mothers and infants facing adversity, such as intimate partner violence, show positive adrenocortical reciprocity (Hibel et al. 2009). Parenting stress has been shown to negatively impact the mother–infant relationship (e.g., Nordahl et al. 2020) and to predict HPA axis dysregulation in early childhood (Wesarg et al. 2020). These findings suggest that parenting stress may hinder mother–infant dyads from forming strong bonds that facilitate effective dyadic stress regulation across early development. Although beyond the scope of the current study, it is plausible that mothers’ physiological activation during stress enables differing dyadic cascades depending on the mothers’ experience of parenting more generally; in particular, those reporting low parenting stress may be successfully mobilizing to help downregulate their infant's stress, whereas those reporting high parenting stress may be unable to do so. Supporting this hypothesis, existing literature indicates that the implications of elevated maternal cortisol for infant stress vary depending on mothers’ psychological functioning, with infants of mothers with high cortisol only showing elevated cortisol responses when the mother also had high depressive symptoms (Khoury et al. 2016). This hypothesis further aligns with our previous findings that increases in infant cortisol reactivity over time were observed only among those whose mothers experienced more depressive symptoms in the early postpartum (H. K. Laurent et al. 2017), and mothers with escalating postpartum depressive symptoms themselves showed elevated cortisol during stress by 18 months postnatal (H. Laurent et al. 2018). Taken together, these findings suggest that these more stressed/distressed mothers’ behavioral responding may result in an upward shift in the infant's cortisol levels while failing to sufficiently downregulate their own physiological reactivity—leading to “unwittingly and unfavorably attuned” dyadic cortisol levels (Atkinson et al. 2016).

In addition to these main effects, we found a moderated effect of mindful parenting on mother–infant cortisol reciprocity such that under the majority of stress conditions (bottom 60%), mindfulness predicted inverse reciprocity, whereas at the highest levels of parenting stress (top 9%), mindfulness predicted positive reciprocity. Although this moderation effect should be interpreted with caution due to the relatively small sample size, it may suggest that in dyads where mothers reported lower parenting stress, mindfulness may have supported mothers in effectively facilitating downregulation of their infants’ stress responses over time, as argued above. Under the high‐stress conditions, on the other hand, mother–infant physiological reciprocity may enable primary caregivers to better assist their children in regulating emotions by enhancing parents’ awareness, a fundamental component of mindfulness (Borelli et al. 2019). In highly stressful situations, it becomes especially critical for the parent to recognize and respond to the infant's emotional needs, and mothers and children may activate their HPA axes more in parallel as a way to cope with the mutual challenge, even though this pattern is often considered maladaptive in high‐risk samples (Atkinson et al. 2016; H. K. Laurent et al. 2011).

### 4.4 Significance of Examining Reciprocity at Multiple Temporal Levels

To our knowledge, the larger longitudinal study on which the present investigation is based was the first to examine mother–infant reciprocity at both within‐ and across‐episode levels, providing a foundation for understanding this process as a *developmental* phenomenon. This is an important area of inquiry given that infancy and toddlerhood represent developmentally sensitive periods, characterized by a general decrease in HPA axis responsiveness during the first year under normative development—a pattern that was also observed in our study sample in previous research (H. K. Laurent et al. 2017; Tarullo and Gunnar 2006). This pattern of lowered adrenocortical responsiveness is thought to parallel the stress hyporesponsive period commonly observed in rats, and high‐quality parental caregiving is necessary to enable this normative downward shift in both rats and humans (Gunnar et al. 1996; Nachmias et al. 1996; Suchecki et al. 1993). Findings from the present study are consistent with the idea that mothers experiencing higher parenting stress and/or a lower‐quality caregiving history themselves are unable to assist their infants in such developmental downregulation, presumably because both parent and infant maintain high HPA activation without learning to recover across repeated parenting challenges (Tarullo and Gunnar 2006). It will be important to continue examining the conditions for and outcomes associated with cortisol reciprocity both within‐ and across‐episodes, and how these patterns link to mother/infant levels and dynamics of HPA activation themselves, to achieve a more comprehensive understanding of implications for the well‐being of mother–infant dyads.

### 4.5 Limitations and Future Directions for Research

Limitations of the present study provide valuable guidance for future research. As with many similar studies, causal inferences cannot be drawn from the present findings due to the study's non‐experimental design. Future studies should employ intervention designs to examine effects of increasing mindfulness and/or reducing parenting stress on parent–infant cortisol reciprocity to better explore such causal chains. The scope of this study also did not allow us to determine behavioral dimensions of the processes involved in mother–infant reciprocity, including bidirectional relations between physiology and behavior as these evolve over time. As infants can only directly respond to caregivers’ behavior (Feldman 2012), identifying behavioral markers associated with positive versus inverse reciprocity will be important for informing prevention/intervention efforts. Furthermore, the relatively small sample size—with higher dropout of participants at the lower end of the parental care spectrum—reduced the statistical power needed to detect complex multilevel effects and interactions. Indeed, post hoc power analysis indicated that the study was only sufficiently powered to detect large effects, which underscores the need to interpret the findings with caution. Given that lower SES has been associated with elevated parenting stress (Martins et al. 2023), future research should explore pathways from SES to parenting stress to reciprocity—either as independent effects or within a moderated mediation model—that the current study was underpowered to test. These sample limitations may have hindered a comprehensive understanding of how parental care history influences mother–infant physiological reciprocity, particularly for individuals with lower parental care histories. While the reasons for this disparity remain unclear, mothers with lower histories of parental care may have faced additional challenges in maintaining study participation, which raises concerns about the feasibility of similar studies for individuals with more difficult backgrounds. This highlights the need for future research designed to recruit and retain diverse samples that can better capture the influence of contextual factors, including but not limited to parental care history, on cortisol reciprocity in high‐risk populations.

Another limitation has to do with the assessment of mindful parenting and parenting stress at a single timepoint (3 months postpartum). Although this allowed us to prospectively predict the unfolding of mother–infant reciprocity processes based on early perceptions of parenting, relying on a single measure separated in time from the physiological processes under investigation may have hindered detection of more proximal (within‐session) reciprocity effects. Future research could address this by assessing these variables concurrently with each cortisol collection point (i.e., at 6, 12, and 18 months postpartum) to determine whether concurrent associations exist between mindful parenting, parenting stress, and mother–infant reciprocity. In addition, future studies could include infant age in months as a primary Level‐2 predictor to examine how within‐session attunement patterns change over time and how these changes relate to parent and infant characteristics, in order to assess their developmental implications (Hibel et al. 2015). We further note that all participants in this study identified as mothers. As a result, the findings do not offer insight into how maternal mindfulness may relate to caregiver–infant physiological reciprocity among other types of caregivers. Future research should include other caregivers, such as fathers, as prior studies have shown that even when mothers and fathers exhibit similar patterns of physiological reciprocity with their infants, the implications for infants’ experiences can differ (Bader et al. 2021; Feldman 2003). Lastly, all stressors employed in this study were laboratory‐based rather than naturalistic stressors conducted in participants’ home environments. Although the ecological validity of the stress paradigms used—the Still Face Procedure, the Strange Situation, and the Maternal Separation and Stranger Approach episodes—has been well established (e.g., Ainsworth et al. 1978; Lo et al. 2015; Mesman et al. 2009), future research directly comparing mother–infant physiological reciprocity and its relation to maternal mindfulness across laboratory and home settings is warranted.

## 5 Conclusion

The present study offers a significant contribution to the physiological reciprocity literature by shedding light on nuanced relations between maternal mindfulness and mother–infant cortisol reciprocity across a critical early developmental period. Importantly, separating within‐episode from between‐episode developmental reciprocity revealed an inverse association—echoing effects of other positive parenting markers—at the latter level only; this underlines the value of a longitudinal lens to better clarify the implications of dyadic reciprocity in high‐risk populations. These findings add to knowledge of when and how positive versus inverse reciprocity can be considered adaptive and serve as a foundation for further exploration of mindful parenting processes in psychophysiological stress adaptation across differing risk conditions.

## Conflicts of Interest

The authors declare no conflicts of interest.

## Data Availability

Data are available upon reasonable request from the corresponding author.

## References

[dev70153-bib-0001] Ahemaitijiang, N. , H. Fang , Y. Ren , Z. R. Han , and N. N. Singh . 2021. “A Review of Mindful Parenting: Theory, Measurement, Correlates, and Outcomes.” Journal of Pacific Rim Psychology 15: 18344909211037016. 10.1177/18344909211037016.

[dev70153-bib-0102] Ainsworth, M. D. S. , and B. A. Wittig . 1969. “Attachment and Exploratory Behaviour of One‐ year‐olds in a Strange situation.” In Determinants of Infant Behaviour, IV, edited by B. M. Foss (Ed.). London: Methuen. Pp. 111–136.

[dev70153-bib-0104] Ainsworth, M. D. S. , M. C. Blehar , E. Waters , and S. Wall . 1978. Patterns of Attachment: a Psychological Study of the Strange Situation. Lawrence Erlbaum.

[dev70153-bib-0002] Anand, L. , I. Sadowski , M. Per , and B. Khoury . 2023. “Mindful Parenting: A Meta‐Analytic Review of Intrapersonal and Interpersonal Parental Outcomes.” Current Psychology 42, no. 10: 8367–8383. 10.1007/s12144-021-02111-w.

[dev70153-bib-0100] Appelbaum, M. , H. Cooper , R. B. Kline , E. Mayo‐Wilson , A. M. Nezu , and S. M. Rao . 2018. “Journal Article Reporting Standards for Quantitative Research in Psychology: the APA Publications and Communications Board Task Force Report.” American Psychologist 73, no. 1: 3–25. 10.1037/amp0000191. 29345484

[dev70153-bib-0003] Armstrong‐Carter, E. , and E. H. Telzer . 2022. “Biological Sensitivity to Environmental Context Fluctuates Dynamically Within Individuals From Day to Day.” Scientific Reports 12, no. 1: 11134. 10.1038/s41598-022-14481-7. 35778425 PMC9249914

[dev70153-bib-0004] Atkinson, L. , A. Gonzalez , D. A. Kashy , et al. 2013. “Maternal Sensitivity and Infant and Mother Adrenocortical Function Across Challenges.” Psychoneuroendocrinology 38, no. 12: 2943–2951. 10.1016/j.psyneuen.2013.08.001. 24007973

[dev70153-bib-0005] Atkinson, L. , B. Jamieson , J. Khoury , J. Ludmer , and A. Gonzalez . 2016. “Stress Physiology in Infancy and Early Childhood: Cortisol Flexibility, Attunement and Coordination.” Journal of Neuroendocrinology 28, no. 8: 12408. 10.1111/jne.12408. 27344031

[dev70153-bib-0006] Atkinson, L. , J. Khoury , B. Jamieson , J. Nofech‐Mozes , and A. Gonzalez . 2024. “Adrenocortical Reactivity in Infancy and Early Childhood: Allostatic Function as Flexibility, Attunement, and Coordination.” In WAIMH Handbook of Infant and Early Childhood Mental Health: Biopsychosocial Factors, edited by J. D. Osofsky , H. E. Fitzgerald , M. Keren , and K. Puura , 185–204. Springer International Publishing.

[dev70153-bib-0007] Azhari, A. , W. Q. Leck , G. Gabrieli , et al. 2019. “Parenting Stress Undermines Mother‐Child Brain‐to‐Brain Synchrony: A Hyperscanning Study.” Scientific Reports 9, no. 1: 11407. 10.1038/s41598-019-47810-4. 31388049 PMC6684640

[dev70153-bib-0008] Bader, L. R. , L. Tan , R. Gonzalez , et al. 2021. “Adrenocortical Interdependence in Father‐Infant and Mother‐Infant Dyads: Attunement or Something More?” Developmental Psychobiology 63, no. 5: 1534–1548. 10.1002/dev.22110. 33615462 PMC8856509

[dev70153-bib-0009] Baer, R. A. , G. T. Smith , J. Hopkins , J. Krietemeyer , and L. Toney . 2006. “Using Self‐Report Assessment Methods to Explore Facets of Mindfulness.” Assessment 13, no. 1: 27–45. 10.1177/1073191105283504. 16443717

[dev70153-bib-0087] van Bakel, H. J. A. , and J. M. Riksen‐Walraven . 2008. “Adrenocortical and Behavioral Attunement in Parents With 1‐Year‐Old Infants.” Developmental Psychobiology 50, no. 2: 196–201. 10.1002/dev.20281. 18286586

[dev70153-bib-0093] Beebe, B. , J. Jaffe , S. Markese , et al. 2010. “The Origins of 12‐Month Attachment: a Microanalysis of 4‐Month Mother‐Infant Interaction.” Attachment & Human Development 12, no. 0: 3–141. 10.1080/14616730903338985. 20390524 PMC3763737

[dev70153-bib-0010] Beeghly, M. , and E. Tronick . 2011. “Early Resilience in the Context of Parent‐Infant Relationships: A Social Developmental Perspective.” Current Problems in Pediatric and Adolescent Health Care 41, no. 7: 197–201. 10.1016/j.cppeds.2011.02.005. 21757137 PMC3137799

[dev70153-bib-0011] Bell, M. A. 2020. “Mother‐Child Behavioral and Physiological Synchrony.” In Advances in Child Development and Behavior, 163–188. Elsevier. 10.1016/bs.acdb.2020.01.006. 32169195

[dev70153-bib-0012] Bernard, N. K. , D. A. Kashy , A. A. Levendosky , G. A. Bogat , and J. S. Lonstein . 2017. “Do Different Data Analytic Approaches Generate Discrepant Findings When Measuring Mother–Infant HPA Axis Attunement?” Developmental Psychobiology 59, no. 2: 174–184. 10.1002/dev.21474. 27966217

[dev70153-bib-0107] Berry, J. O. , and W. H. Jones . 1995. “The Parental Stress Scale: Initial Psychometric Evidence.” Journal of Social and Personal Relationships 12, no. 3: 463–472. 10.1177/0265407595123009.

[dev70153-bib-0013] Borelli, J. L. , D. Shai , P. A. Smiley , et al. 2019. “Mother–Child Adrenocortical Synchrony: Roles of Maternal Overcontrol and Child Developmental Phase.” Developmental Psychobiology 61, no. 8: 1120–1134. 10.1002/dev.21845. 30868558

[dev70153-bib-0014] Bornstein, M. H. 2012. “Cultural Approaches to Parenting.” Parenting: Science and Practice 12, no. 2–3: 212–221. 10.1080/15295192.2012.683359. 22962544 PMC3433059

[dev70153-bib-0015] Bornstein, M. H. 2022. “Mother‐Infant Attunement: A Multilevel Approach via Body, Brain, and Behavior.” In Parenting: Selected Writings of Marc H. Bornstein, 280–315. Routledge.

[dev70153-bib-0016] Bowlby, J. 1969. Attachment and Loss, Vol. 1: Attachment. Basic Books.

[dev70153-bib-0017] Boyce, W. T. , and B. J. Ellis . 2005. “Biological Sensitivity to Context: I. An Evolutionary‐Developmental Theory of the Origins and Functions of Stress Reactivity.” Development and Psychopathology 17, no. 2: 271–301. 10.1017/s0954579405050145. 16761546

[dev70153-bib-0018] Brazelton, T. B. , E. Tronick , L. Adamson , H. Als , and S. Wise . 1975. “Early Mother‐Infant Reciprocity.” In Ciba Foundation Symposium 33—Parent‐Infant Interaction, edited by R. Porter and M. O'Connor , 137–154. John Wiley & Sons, Ltd. 10.1002/9780470720158.ch9 1045978

[dev70153-bib-0019] Broesch, T. , E. E. Little , L. J. Carver , and C. H. Legare . 2022. “Still‐Face Redux: Infant Responses to a Classic and Modified Still‐Face Paradigm in Proximal and Distal Care Cultures.” Infant Behavior & Development 68: 101732. 10.1016/j.infbeh.2022.101732. 35760032

[dev70153-bib-0020] Buhler‐Wassmann, A. C. , and L. C. Hibel . 2021. “Studying Caregiver‐Infant Co‐Regulation in Dynamic, Diverse Cultural Contexts: A Call to Action.” Infant Behavior & Development 64: 101586. 10.1016/j.infbeh.2021.101586. 34118652 PMC10314734

[dev70153-bib-0021] Burzler, M. A. , and U. S. Tran . 2022. “Dispositional Mindfulness and the Process of Mindfulness Cultivation: A Qualitative Synthesis and Critical Assessment of the Extant Literature on the Five Facet Mindfulness Questionnaire (FFMQ).” Collabra: Psychology 8, no. 1: 56176. 10.1525/collabra.56176.

[dev70153-bib-0022] Campbell, K. , J. W. Thoburn , and H. D. Leonard . 2017. “The Mediating Effects of Stress on the Relationship Between Mindfulness and Parental Responsiveness.” Couple and Family Psychology: Research and Practice 6, no. 1: 48–59. 10.1037/cfp0000075.

[dev70153-bib-0023] Choi, J. , H. K. Kim , D. M. Capaldi , and J. J. Snodgrass . 2021. “Long‐Term Effects of Father Involvement in Childhood on Their Son's Physiological Stress Regulation System in Adulthood.” Developmental Psychobiology 63, no. 6: e22152. 10.1002/dev.22152. 34124784 PMC8923429

[dev70153-bib-0024] Cristóbal Cañadas, D. , T. Parrón Carreño , C. Sánchez Borja , and A. Bonillo Perales . 2022. “Benefits of Kangaroo Mother Care on the Physiological Stress Parameters of Preterm Infants and Mothers in Neonatal Intensive Care.” International Journal of Environmental Research and Public Health 19, no. 12: 7183. 10.3390/ijerph19127183. 35742429 PMC9223087

[dev70153-bib-0025] Crockett, E. E. , B. M. Holmes , D. A. Granger , and K. Lyons‐Ruth . 2013. “Maternal Disrupted Communication During Face‐to‐Face Interaction at 4 Months: Relation to Maternal and Infant Cortisol Among At‐Risk Families.” Infancy: The Official Journal of the International Society on Infant Studies 18, no. 6: 1111–1134. 10.1111/infa.12015. 25506272 PMC4264526

[dev70153-bib-0026] Davis, M. , K. West , J. Bilms , D. Morelen , and C. Suveg . 2018. “A Systematic Review of Parent–Child Synchrony: It Is More Than Skin Deep.” Developmental Psychobiology 60, no. 6: 674–691. 10.1002/dev.21743. 29900545

[dev70153-bib-0028] DePasquale, C. E. 2020. “A Systematic Review of Caregiver–Child Physiological Synchrony Across Systems: Associations With Behavior and Child Functioning.” Development and Psychopathology 32, no. 5: 1754–1777. 10.1017/S0954579420001236. 33427185

[dev70153-bib-0029] DiCorcia, J. A. , and E. Tronick . 2011. “Quotidian Resilience: Exploring Mechanisms That Drive Resilience From a Perspective of Everyday Stress and Coping.” Neuroscience and Biobehavioral Reviews 35, no. 7: 1593–1602. 10.1016/j.neubiorev.2011.04.008. 21513731

[dev70153-bib-0030] Doiron, K. M. , D. M. Stack , D. J. Dickson , S. Bouchard , and L. A. Serbin . 2022. “Co‐Regulation and Parenting Stress Over Time in Full‐Term, Very Low Birthweight Preterm, And Psycho‐Socially At‐Risk Infant‐Mother Dyads: Implications for Fostering the Development of Healthy Relationships.” Infant Behavior and Development 68: 101731. 10.1016/j.infbeh.2022.101731. 35850046

[dev70153-bib-0031] Duncan, L. G. , J. D. Coatsworth , and M. T. Greenberg . 2009. “A Model of Mindful Parenting: Implications for Parent–Child Relationships and Prevention Research.” Clinical Child and Family Psychology Review 12, no. 3: 255–270. 10.1007/s10567-009-0046-3. 19412664 PMC2730447

[dev70153-bib-0106] Duncan, L. G. 2007. Assessment of Mindful Parenting Among Families of Earlyadolescents: Development and Validation of the Interpersonal Mindful‐ness in Parenting Scale. Unpublished doctoral dissertation. PennsylvaniaState University.

[dev70153-bib-0032] Falkenström, F. , O. A. Solbakken , C. Möller , B. Lech , R. Sandell , and R. Holmqvist . 2014. “Reflective Functioning, Affect Consciousness, and Mindfulness: Are These Different Functions?” Psychoanalytic Psychology 31, no. 1: 26–40. 10.1037/a0034049.

[dev70153-bib-0096] Perry, R. E. , S. H. Braren , M. Opendak , et al. & Family Life Project Key Investigators . 2020. “Elevated Infant Cortisol Is Necessary but Not Sufficient for Transmission of Environmental Risk to Infant Social Development: Cross‐species Evidence of Mother–infant Physiological Social Transmission.” Development and Psychopathology 32, no. 5: 1696–1714. 10.1017/S0954579420001455. 33427190 PMC8951448

[dev70153-bib-0033] Feldman, R. 2003. “Infant–Mother and Infant–Father Synchrony: The Coregulation of Positive Arousal.” Infant Mental Health Journal: Infancy and Early Childhood 24, no. 1: 1–23. 10.1002/imhj.10041.

[dev70153-bib-0034] Feldman, R. 2007. “Parent–Infant Synchrony and the Construction of Shared Timing; Physiological Precursors, Developmental Outcomes, and Risk Conditions.” Journal of Child Psychology and Psychiatry 48, no. 3–4: 329–354. 10.1111/j.1469-7610.2006.01701.x. 17355401

[dev70153-bib-0035] Feldman, R. 2012. “Bio‐Behavioral Synchrony: A Model for Integrating Biological and Microsocial Behavioral Processes in the Study of Parenting.” Parenting 12, no. 2–3: 154–164. 10.1080/15295192.2012.683342.

[dev70153-bib-0097] Fernandes, D. V. , M. C. Canavarro , and H. Moreira . 2021. “The Role of Mothers' self‐compassion on Mother–infant Bonding During the COVID‐19 Pandemic: a Longitudinal Study Exploring the Mediating Role of Mindful Parenting and Parenting Stress in the Postpartum Period.” Infant Mental Health Journal 42, no. 5: 621–635. 10.1002/imhj.21942. 34407224 PMC8426800

[dev70153-bib-0036] Gallistl, M. , R. Linz , L. M. C. Puhlmann , T. Singer , and V. Engert . 2024. “Evidence for Differential Associations of Distinct Trait Mindfulness Facets With Acute and Chronic Stress.” Psychoneuroendocrinology 166: 107051. 10.1016/j.psyneuen.2024.107051. 38678734

[dev70153-bib-0037] Gilmore, J. H. , F. Shi , S. L. Woolson , et al. 2012. “Longitudinal Development of Cortical and Subcortical Gray Matter From Birth to 2 Years.” Cerebral Cortex 22, no. 11: 2478–2485. 10.1093/cercor/bhr327. 22109543 PMC3464410

[dev70153-bib-0027] del Giudice, M. , B. J. Ellis , and E. A. Shirtcliff . 2011. “The Adaptive Calibration Model of Stress Responsivity.” Neuroscience & Biobehavioral Reviews 35, no. 7: 1562–1592. 10.1016/j.neubiorev.2010.11.007. 21145350 PMC3068241

[dev70153-bib-0103] Goldsmith, H. H. , and M. K. Rothbart . 1991. “Contemporary Instruments for Assessing Early Temperament by Questionnaire and in the Laboratory.” In Explorations in Temperament, (249–272). Springer, Boston, MA. 10.1007/978-1-4899-0643-4_16.

[dev70153-bib-0038] Grant, K.‐A. , A. Bautovich , C. McMahon , N. Reilly , L. Leader , and M.‐P. Austin . 2012. “Parental Care and Control During Childhood: Associations With Maternal Perinatal Mood Disturbance and Parenting Stress.” Archives of Women's Mental Health 15, no. 4: 297–305. 10.1007/s00737-012-0292-0. 22695807

[dev70153-bib-0039] Gunnar, M. R. , L. Brodersen , M. Nachmias , K. Buss , and J. Rigatuso . 1996. “Stress Reactivity and Attachment Security.” Developmental Psychobiology 29, no. 3: 191–204. 10.1002/(SICI)1098-2302(199604)29:3<191::AID-DEV1>3.0.CO;2-M. 8666128

[dev70153-bib-0040] Gunnar, M. R. , and C. L. Cheatham . 2003. “Brain and Behavior Interface: Stress and the Developing Brain.” Infant Mental Health Journal: Infancy and Early Childhood 24, no. 3: 195–211. 10.1002/imhj.10052.

[dev70153-bib-0041] Gunnar, M. R. , and B. Donzella . 2002. “Social Regulation of the Cortisol Levels in Early Human Development.” Psychoneuroendocrinology 27, no. 1–2: 199–220. 10.1016/s0306-4530(01)00045-2. 11750779

[dev70153-bib-0042] Gunnar, M. R. , N. M. Talge , and A. Herrera . 2009. “Stressor Paradigms in Developmental Studies: What Does and Does Not Work to Produce Mean Increases in Salivary Cortisol.” Psychoneuroendocrinology 34, no. 7: 953–967. 10.1016/j.psyneuen.2009.02.010. 19321267 PMC2692557

[dev70153-bib-0110] Gunnar, M. R. , L. Brodersen , K. Krueger , and J. Rigatuso . 1996. “Dampening of Adrenocortical Responses During Infancy: Normative Changes and Individual Differences.” Child Development 67, no. 3: 877–889. 8706532

[dev70153-bib-0043] Guo, Y. , S.‐Y. Leu , K. E. Barnard , E. A. Thompson , and S. J. Spieker . 2015. “An Examination of Changes in Emotion Co‐Regulation Among Mother and Child Dyads During the Strange Situation.” Infant and Child Development 24, no. 3: 256–273. 10.1002/icd.1917. 26726296 PMC4694580

[dev70153-bib-0094] Halevi, G. , A. Djalovski , Y. Kanat‐Maymon , et al. 2017. “The Social Transmission of Risk: Maternal Stress Physiology, Synchronous Parenting, and Well‐being Mediate the Effects of War Exposure on Child Psychopathology.” Journal of Abnormal Psychology 126, no. 8: 1087–1103. 10.1037/abn0000307. 29154569

[dev70153-bib-0044] Hibel, L. C. , D. A. Granger , C. Blair , M. J. Cox , and The Family Life Project Key Investigators. 2009. “Intimate Partner Violence Moderates the Association Between Mother–Infant Adrenocortical Activity Across an Emotional Challenge.” Journal of Family Psychology 23, no. 5: 615–625. 10.1037/a0016323. 19803598

[dev70153-bib-0045] Hibel, L. C. , D. A. Granger , C. Blair , E. D. Finegood , and The Family Life Project Key Investigators. 2015. “Maternal‐Child Adrenocortical Attunement in Early Childhood: Continuity and Change.” Developmental Psychobiology 57, no. 1: 83–95. 10.1002/dev.21266. 25417896 PMC5317045

[dev70153-bib-0095] Hibel, L. C. , E. Mercado , and K. Valentino . 2019. “Child Maltreatment and Mother‐child Attunement and Transmission of Stress Physiology.” Child Maltreatment 24, no. 4: 340–352. 10.1177/1077559519826295. 30700154 PMC6710153

[dev70153-bib-0099] Hicks, L. M. , C. J. Dayton , S. Brown , M. Muzik , and H. Raveau . 2018. “Mindfulness Moderates Depression and Quality of Prenatal Attachment in Expectant Parents.” Mindfulness 9, no. 5: 1604–1614. 10.1007/s12671-018-0907-2.

[dev70153-bib-0046] Hostinar, C. E. , R. M. Sullivan , and M. R. Gunnar . 2014. “Psychobiological Mechanisms Underlying the Social Buffering of the HPA Axis: A Review of Animal Models and Human Studies Across Development.” Psychological Bulletin 140, no. 1: 256–282. 10.1037/a0032671. 23607429 PMC3844011

[dev70153-bib-0047] Huynh, T. , M. L. Kerr , C. N. Kim , E. Fourianalistyawati , V. Y.‐R. Chang , and L. G. Duncan . 2024. “Parental Reflective Capacities: A Scoping Review of Mindful Parenting and Parental Reflective Functioning.” Mindfulness 15, no. 7: 1531–1602. 10.1007/s12671-024-02379-6. 39328292 PMC11426413

[dev70153-bib-0048] Kabat‐Zinn, J. 1990. Full Catastrophe Living: Using the Wisdom of Your Body and Mind to Face Stress, Pain, and Illness. Delacorte Press.

[dev70153-bib-0049] Kabat‐Zinn, J. 1994. Wherever You Go, There You are: Mindfulness Meditation in Everyday Life. 1st ed. Hyperion.

[dev70153-bib-0050] Khoury, B. , V. Manova , L. Adel , et al. 2023. “Tri‐Process Model of Interpersonal Mindfulness: Theoretical Framework and Study Protocol.” Frontiers in Psychology 14: 1130959. 10.3389/fpsyg.2023.1130959. 37179876 PMC10170994

[dev70153-bib-0109] Khoury, J. E. , A. Gonzalez , R. Levitan , M. Masellis , V. Basile , and L. Atkinson . 2016. “Maternal Self‐reported Depressive Symptoms and Maternal Cortisol Levels Interact to Predict Infant Cortisol Levels.” Infant Mental Health Journal: Infancy and Early Childhood 37, no. 2: 125–139. 10.1002/imhj.21554. 26939829

[dev70153-bib-0051] Laurent, H. K. , J. C. Ablow , and J. Measelle . 2011. “Risky Shifts: How the Timing and Course of Mothers' Depressive Symptoms Across the Perinatal Period Shape Their Own and Infant's Stress Response Profiles.” Development and Psychopathology 23, no. 2: 521–538. 10.1017/S0954579411000083. 23786693

[dev70153-bib-0053] Laurent, H. K. , L. G. Duncan , A. Lightcap , and F. Khan . 2017. “Mindful Parenting Predicts Mothers' and Infants' Hypothalamic‐Pituitary‐Adrenal Activity During a Dyadic Stressor.” Developmental Psychology 53, no. 3: 417–424. 10.1037/dev0000258. 27893234 PMC13352588

[dev70153-bib-0054] Laurent, H. , S. H. Goodman , Z. N. Stowe , et al. 2018. “Course of Ante‐ and Postnatal Depressive Symptoms Related to Mothers' HPA Axis Regulation.” Journal of Abnormal Psychology 127, no. 4: 404–416. 10.1037/abn0000348. 29745705

[dev70153-bib-0055] Laurent, H. K. , G. T. Harold , L. Leve , K. H. Shelton , and S. H. M. V. Goozen . 2016. “Understanding the Unfolding of Stress Regulation in Infants.” Development and Psychopathology 28, no. 4 pt. 2: 1431. 10.1017/S0954579416000171. 27020470 PMC5301455

[dev70153-bib-0056] Laurent, H. 2017. “Early Calibration of the HPA Axis by Maternal Psychopathology.” Psychoneuroendocrinology 78: 177–184. 10.1016/j.psyneuen.2017.01.034. 28212519

[dev70153-bib-0057] Laurent, H. K. , M. Sbrilli , D. Dawson , M. Finnegan , and D. Ramdas‐Neal . 2021. “Disentangling Levels of Mother–Infant Neuroendocrine Attunement and Longitudinal Relations With Maternal Risk and Protective Factors.” Developmental Psychobiology 63, no. 1: 88–97. 10.1002/dev.21997. 32476146

[dev70153-bib-0058] Leclère, C. , S. Viaux , M. Avril , et al. 2014. “Why Synchrony Matters During Mother‐Child Interactions: A Systematic Review.” PLoS ONE 9, no. 12: e113571. 10.1371/journal.pone.0113571. 25469637 PMC4254467

[dev70153-bib-0105] Lo, S. L. , L. N. Vroman , and C. E. Durbin . 2015. “Ecological Validity of Laboratory Assessments of Child Temperament: Evidence From Parent Perspectives.” Psychological Assessment 27, no. 1: 280–290. 10.1037/pas0000033. 25330108

[dev70153-bib-0098] Madden, V. , J. Domoney , K. Aumayer , et al. 2015. “Intergenerational Transmission of Parenting: Findings From a UK Longitudinal Study.” The European Journal of Public Health 25, no. 6: 1030–1035. 10.1093/eurpub/ckv093. 26037954 PMC4668327

[dev70153-bib-0060] Markovic, V. M. , Z. Cupic , V. Vukojevic , and L. Kolar‐Anic . 2011. “Predictive Modeling of the Hypothalamic‐Pituitary‐Adrenal (HPA) Axis Response to Acute and Chronic Stress.” Endocrine Journal 58, no. 10: 889–904. 10.1507/endocrj.EJ11-0037. 21852742

[dev70153-bib-0061] Martins, P. C. , C. D. Matos , and A. I. Sani . 2023. “Parental Stress and Risk of Child Abuse: The Role of Socioeconomic Status.” Children and Youth Services Review 148: 106879. 10.1016/j.childyouth.2023.106879.

[dev70153-bib-0062] Mchale, J. P. 2007. “When Infants Grow Up in Multiperson Relationship Systems.” Infant Mental Health Journal: Infancy and Early Childhood 28, no. 4: 370–392. 10.1002/imhj.20142. PMC3079566 21512615

[dev70153-bib-0063] McMahon, C. A. , and E. Meins . 2012. “Mind‐Mindedness, Parenting Stress, and Emotional Availability in Mothers of Preschoolers.” Early Childhood Research Quarterly 27, no. 2: 245–252. 10.1016/j.ecresq.2011.08.002.

[dev70153-bib-0064] Mera, S. , M. J. Zimmer‐Gembeck , and E. G. Conlon . 2023. “Emerging Adults' Experience of Mindful Parenting: Distinct Associations With Their Dispositional and Interpersonal Mindfulness, Self‐Compassion, and Adjustment.” Emerging Adulthood 11, no. 5: 1180–1195. 10.1177/21676968231185888.

[dev70153-bib-0065] Mesman, J. , M. H. van IJzendoorn , and M. J. Bakermans‐Kranenburg . 2009. “The Many Faces of the Still‐Face Paradigm: A Review and Meta‐Analysis.” Developmental Review 29, no. 2: 120–162. 10.1016/j.dr.2009.02.001.

[dev70153-bib-0066] Nachmias, M. , M. Gunnar , S. Mangelsdorf , R. H. Parritz , and K. Buss . 1996. “Behavioral Inhibition and Stress Reactivity: The Moderating Role of Attachment Security.” Child Development 67, no. 2: 508–522. 10.1111/j.1467-8624.1996.tb01748.x. 8625725

[dev70153-bib-0067] Nofech‐Mozes, J. A. L. , B. Jamieson , A. Gonzalez , and L. Atkinson . 2020. “Mother–Infant Cortisol Attunement: Associations With Mother–Infant Attachment Disorganization.” Development and Psychopathology 32, no. 1: 43–55. 10.1017/S0954579418001396. 30636650

[dev70153-bib-0068] Nordahl, D. , K. Rognmo , A. Bohne , et al. 2020. “Adult Attachment Style and Maternal‐Infant Bonding: The Indirect Path of Parenting Stress.” BMC Psychology 8, no. 1: 58. 10.1186/s40359-020-00424-2. 32513300 PMC7278048

[dev70153-bib-0069] Obradović, J. , X. A. Portilla , and P. J. Ballard . 2016. “Biological Sensitivity to Family Income: Differential Effects on Early Executive Functioning.” Child Development 87, no. 2: 374–384. 10.1111/cdev.12475. 26709089

[dev70153-bib-0070] Parent, J. , J. Clifton , R. Forehand , A. Golub , M. Reid , and E. R. Pichler . 2014. “Parental Mindfulness and Dyadic Relationship Quality in Low‐Income Cohabiting Black Stepfamilies: Associations With Parenting Experienced by Adolescents.” Couple & Family Psychology 3, no. 2: 67–82. 10.1037/cfp0000020. 25544936 PMC4274993

[dev70153-bib-0071] Parker, G. , H. Tupling , and L. B. Brown . 1979. “A Parental Bonding Instrument.” British Journal of Medical Psychology 52, no. 1: 1–10. 10.1111/j.2044-8341.1979.tb02487.x.

[dev70153-bib-0072] Passaquindici, I. , M. Pastore , O. Nardozza , et al. 2024. “From Inner to Dyadic Connection: The Role of Mindfulness in Mother–Infant Interaction During the First Year of Life.” Frontiers in Behavioral Neuroscience 18: 1398042. 10.3389/fnbeh.2024.1398042. 39176254 PMC11338867

[dev70153-bib-0073] Pratt, M. , Y. Apter‐Levi , A. Vakart , Y. Kanat‐Maymon , O. Zagoory‐Sharon , and R. Feldman . 2017. “Mother‐Child Adrenocortical Synchrony; Moderation by Dyadic Relational Behavior.” Hormones and Behavior 89: 167–175. 10.1016/j.yhbeh.2017.01.003. 28131596

[dev70153-bib-0108] Preacher, K. J. , P. J. Curran , and D. J. Bauer . 2006. “Computational Tools for Probing Interactions in Multiple Linear Regression, Multilevel Modeling, and Latent Curve Analysis.” Journal of Educational and Behavioral Statistics 31, no. 4: 437–448. 10.3102/10769986031004437.

[dev70153-bib-0074] Provenzi, L. , G. Scotto di Minico , L. Giusti , E. Guida , and M. Müller . 2018. “Disentangling the Dyadic Dance: Theoretical, Methodological and Outcomes Systematic Review of Mother‐Infant Dyadic Processes.” Frontiers in Psychology 9: 348. 10.3389/fpsyg.2018.00348. 29615947 PMC5868133

[dev70153-bib-0075] Raudenbush, S. W. , A. S. Bryk , Y. F. Cheong , R. T. Congdon Jr , and M. D. Toit . 2016. HLM7 Hierarchical Linear and Nonlinear Modeling User Manual: User Guide for Scientific Software International's (S.S.I.) Program. Scientific Software International, Incorporated.

[dev70153-bib-0076] Rivera, C. E. , L. W. Coyne , K. M. Daigle , A. Guzick , A. Reid , and S. Shea . 2022. “Mindfulness, Parenting Behavior, and Children's Mental Health: An Investigation Among Diverse, Low‐Income Mothers of Preschool Aged Children.” Journal of Contextual Behavioral Science 24: 79–86. 10.1016/j.jcbs.2022.03.003.

[dev70153-bib-0078] Sansone, A. 2024. “The Central Role of Mindful Parenting in Child's Emotional Regulation and Human Flourishing: A Blueprint Perspective.” Frontiers in Psychology 15: 1420588. 10.3389/fpsyg.2024.1420588. 38988375 PMC11233750

[dev70153-bib-0079] Shaver, P. R. , S. Lavy , C. D. Saron , and M. Mikulincer . 2007. “Social Foundations of the Capacity for Mindfulness: An Attachment Perspective.” Psychological Inquiry 18, no. 4: 264–271. 10.1080/10478400701598389.

[dev70153-bib-0080] Skoranski, A. , J. D. Coatsworth , and E. Lunkenheimer . 2019. “A Dynamic Systems Approach to Understanding Mindfulness in Interpersonal Relationships.” Journal of Child and Family Studies 28, no. 10: 2659–2672. 10.1007/s10826-019-01500-x.

[dev70153-bib-0081] Smith, S. M. , and W. W. Vale . 2006. “The Role of the Hypothalamic‐Pituitary‐Adrenal Axis in Neuroendocrine Responses to Stress.” Dialogues in Clinical Neuroscience 8, no. 4: 383–395. 10.31887/DCNS.2006.8.4/ssmith. 17290797 PMC3181830

[dev70153-bib-0082] Stallings, J. , A. S. Fleming , C. Corter , C. Worthman , and M. Steiner . 2001. “The Effects of Infant Cries and Odors on Sympathy, Cortisol, and Autonomic Responses in New Mothers and Nonpostpartum Women.” Parenting 1, no. 1–2: 71–100. 10.1080/15295192.2001.9681212.

[dev70153-bib-0083] Suchecki, D. , P. Rosenfeld , and S. Levine . 1993. “Maternal Regulation of the Hypothalamic‐Pituitary‐Adrenal Axis in the Infant Rat: The Roles of Feeding and Stroking.” Developmental Brain Research 75, no. 2: 185–192. 10.1016/0165-3806(93)90022-3. 8261610

[dev70153-bib-0084] Tarullo, A. R. , and M. R. Gunnar . 2006. “Child Maltreatment and the Developing HPA Axis.” Hormones and Behavior, Translational Topics in Behavioral Neuroendocrinology 50, no. 4: 632–639. 10.1016/j.yhbeh.2006.06.010. 16876168

[dev70153-bib-0101] Toda, S. , and A. Fogel . 1993. “Infant Response to the Still‐face Situation at 3 and 6 Months.” Developmental Psychology 29, no. 3: 532–538. 10.1037/0012-1649.29.3.532.

[dev70153-bib-0085] Townshend, K. 2016. “Conceptualizing the Key Processes of Mindful Parenting and Its Application to Youth Mental Health.” Australasian Psychiatry 24, no. 6: 575–577. 10.1177/1039856216654392. 27354336

[dev70153-bib-0086] Troller‐Renfree, S. V. , N. H. Brito , P. M. Desai , et al. 2020. “Infants of Mothers With Higher Physiological Stress Show Alterations in Brain Function.” Developmental Science 23, no. 6: e12976. 10.1111/desc.12976. 32329125 PMC9126078

[dev70153-bib-0092] Tronick, E. 2007. The Neurobehavioral and Social‐emotional Development of Infants and Children, (xii, 571). W. W. Norton & Company.

[dev70153-bib-0088] Vargas‐Uricoechea, H. , A. Castellanos‐Pinedo , K. Urrego‐Noguera , et al. 2024. “Mindfulness‐Based Interventions and the Hypothalamic–Pituitary–Adrenal Axis: A Systematic Review.” Neurology International 16, no. 6: 1552–1584. 10.3390/neurolint16060115. 39585074 PMC11587421

[dev70153-bib-0089] Vernon, J. R. G. , and M. M. Moretti . 2024. “Parent Emotion Regulation, Mindful Parenting, and Youth Attachment: Direct and Indirect Associations With Internalizing and Externalizing Problems.” Child Psychiatry & Human Development 55, no. 4: 987–998. 10.1007/s10578-022-01446-0. 36322236

[dev70153-bib-0090] Wesarg, C. , A. L. Van Den Akker , N. Y. L. Oei , M. Hoeve , and R. W. Wiers . 2020. “Identifying Pathways From Early Adversity to Psychopathology: A Review on Dysregulated HPA Axis Functioning and Impaired Self‐regulation in Early Childhood.” European Journal of Developmental Psychology 17, no. 6: 808–827. 10.1080/17405629.2020.1748594.

[dev70153-bib-0091] Yan, J. J. , S. Schoppe‐Sullivan , Q. Wu , and Z. R. Han . 2021. “Associations From Parental Mindfulness and Emotion Regulation to Child Emotion Regulation Through Parenting: The Moderating Role of Coparenting in Chinese Families.” Mindfulness 12, no. 6: 1513–1523. 10.1007/s12671-021-01619-3.

[dev70153-bib-0098a] Fernandes, D. V. , A. R. Martins , M. C. Canavarro , and H. Moreira . 2022. “Mindfulness‐ and Compassion‐Based Parenting Interventions Applied to the Postpartum Period: A Systematic Review.” Journal of Child and Family Studies 31, no. 2: 563–587. 10.1007/s10826-021-02175-z.

